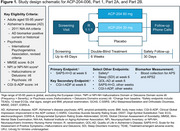# Plasma Biomarker Characterization of Alzheimer’s Disease Psychosis in Study ACP‐204‐006

**DOI:** 10.1002/alz70859_106556

**Published:** 2025-12-26

**Authors:** Bryan Dirks, Samantha M Friend, Becky M Howell, Christine M Murphy, Peter M Zhang, Xiaoshu Feng, Sanjeev S Pathak

**Affiliations:** ^1^ Acadia Pharmaceuticals Inc., Princeton, NJ USA; ^2^ Acadia Pharmaceuticals Inc., San Diego, CA USA

## Abstract

**Background:**

Alzheimer’s disease (AD) is diagnosed and staged based on biological criteria that utilize biomarkers. However, biomarker data are sparse in patients with psychosis and significant cognitive decline. Here we present screening metrics based on study biomarker requirements in patients diagnosed using 2011 National Institute on Aging‐Alzheimer Association (NIA‐AA) criteria for AD and International Psychogeriatrics Association (IPA) criteria for psychosis. Patients were enrolled in the context of the ACP‐204 Alzheimer’s disease psychosis (ADP) clinical development program. ACP‐204 is a potent inverse agonist and antagonist of serotonin 2A (5‐HT2A) receptors.

**Methods:**

ACP‐204‐006 is intended to enroll patients with ADP into a master protocol [ACP‐204‐006 (NCT06159673)]. This includes Part 1, one phase 2 efficacy and dose‐confirming study, and Parts 2A and 2B, two independent phase 3 confirmatory efficacy studies, for ACP‐204 in ADP. Each study has the same design (6‐week double‐blind treatment); patients may participate in only a single Part (Figure 1). In addition to meeting clinical criteria for ADP, participants must have confirmed plasma or historical biomarker positivity. Blood samples collected at screening were tested for Amyloid Probability Score (APS), APS2, and phosphorylated tau at threonine 217 (p‐tau217) ratio using commercially available blood tests with diagnostic algorithms to predict amyloid brain status. These include amyloid beta (Aβ)42/40 ratios and p‐tau217 ratios.

**Results:**

In Part 1 (currently ongoing), between February 6, 2024, and December 27, 2024, 322 adults were screened, of whom 75 (aged 59‐89 years) met NIA‐AA and IPA criteria. Of these, 23 met clinical criteria for ADP but were not positive for APS or APS2, despite meeting historical biomarker requirements. We will present detailed biomarker results on the population, including apolipoprotein E (apoE) proteotype, Aβ42/40 ratio, and p‐tau217 ratio. Clinical presentation and related information will be detailed as well.

**Conclusions:**

Biomarkers, rather than syndromic presentation, are recommended as a mechanism by which to define AD biologically. Since biomarker data from patients presenting with clinical symptoms consistent with Alzheimer’s disease dementia and psychosis are sparse, we present biomarker data in a rigorously phenotyped population. These results can also inform the design and execution of clinical trials in ADP in the future.